# Endogenous Long Pentraxin 3 Exerts a Protective Role in a Murine Model of Pulmonary Fibrosis

**DOI:** 10.3389/fimmu.2021.617671

**Published:** 2021-02-18

**Authors:** Federica Maccarinelli, Mattia Bugatti, Ander Churruca Schuind, Sara Ganzerla, William Vermi, Marco Presta, Roberto Ronca

**Affiliations:** ^1^Department of Molecular and Translational Medicine, University of Brescia, Brescia, Italy; ^2^ASST Spedali Civili di Brescia, Brescia, Italy

**Keywords:** long pentraxin-3, lung fibrosis, bleomycin, stroma, fibroblast, immune infiltrate

## Abstract

Pulmonary fibrosis is a progressive scarring disease of the lungs, characterized by inflammation, fibroblast activation, and deposition of extracellular matrix. The long pentraxin 3 (PTX3) is a member of the pentraxin family with non-redundant functions in innate immune responses, tissue repair, and haemostasis. The role played in the lungs by PTX3 during the fibrotic process has not been elucidated. In this study, the impact of PTX3 expression on lung fibrosis was assessed in an intratracheal bleomycin (BLM)-induced murine model of the disease applied to wild type animals, transgenic mice characterized by endothelial overexpression and stromal accumulation of PTX3 (Tie2-PTX3 mice), and genetically deficient *Ptx3*^−/−^ animals. Our data demonstrate that PTX3 is produced during BLM-induced fibrosis in wild type mice, and that PTX3 accumulation in the stroma compartment of Tie2-PTX3 mice limits the formation of fibrotic tissue in the lungs, with reduced fibroblast activation and collagen deposition, and a decrease in the recruitment of the immune infiltrate. Conversely, *Ptx3*-null mice showed an exacerbated fibrotic response and decreased survival in response to BLM treatment. These results underline the protective role of endogenous PTX3 during lung fibrosis and pave the way for the study of novel PTX3-derived therapeutic approaches to the disease.

## Introduction

Pulmonary fibrosis (PF) includes more than 200 different pathological conditions characterized by inflammation and scar tissue formation in the lungs. PF can be grouped in five categories: drug-induced, radiation-induced, environmental, autoimmune, and occupational (1). However, the most common type of PF is represented by the “Idiopathic Pulmonary Fibrosis” (IPF), whose etiology remains unknown. In the last years, the incidence of IPF has increased over time in most countries worldwide, with approximately 50,000 new cases diagnosed each year in the U.S (2).

Starting symptoms of IPF, such as shortness of breath, dry hacking cough and fatigue, get significantly worse when the deposition of scar tissue augments, increases in stiffness and causes an irreversible loss of pulmonary functionality (3).

Despite the unknown cause(s) of IPF, aging (IPF is rare before 50 years of age), cigarette smoking, and genetic predisposition may represent relevant risk factors (4, 5). At histopathological level, IPF appears as a progressive scarring disease in the lungs, characterized by injury and hyperplasia of alveolar epithelial cells and fibroblasts, accumulation of inflammatory cells, consistent deposition of extracellular matrix (ECM), and formation of scars (6). Particular attention has been given to the onset of an inflammatory response and to the presence of an immune infiltrate that establishes and sustains a damaging and fibrotic context in the lungs (7). Even though little is known about the role of monocytes and tissue-resident macrophages in lung fibrosis (8), macrophages are persistently increased in the lung in close proximity to collagen-producing fibroblasts, in keeping with the role exerted by monocytes in the development of experimental fibrosis (9, 10). In particular, M2 macrophages accumulating in the lungs during fibrogenesis have been identified as the major source of several pro-fibrotic mediators, which stimulate fibroblast proliferation and collagen synthesis/deposition (11–14).

Different *in vivo* animal models of PF have been developed to understand the evolution of fibrotic responses in the lungs and in other organs (15), allowing the identification of cell types, mediators, and processes that are likely involved also in the human disease. To date, bleomycin (BLM)-induced pulmonary fibrosis in mice represents the best characterized murine model to study PF and IPF (15, 16). BLM is an antibiotic efficacious for the treatment of squamous cell carcinomas and skin tumors, but with limiting and dose-dependent pulmonary toxicity that results in progressive fibrosis (17). In mice, a single (or multiple, according to the specific schedule) intratracheal administration of BLM causes lung injury, resulting in pulmonary fibrosis that can be observed by day 14 (15, 18–20). The initial direct damage to alveolar epithelial cells is followed by dense ECM deposition associated with vessel remodeling and intense inflammatory infiltration, mimicking what is observed in patients (15).

The soluble pattern recognition receptor long pentraxin-3 (PTX3) is a member of the pentraxin family and a component of the humoral arm of the innate immunity. PTX3 expression is normally low in tissues and serum under physiological conditions, but the levels of PTX3 quickly rise in the presence of inflammatory and/or infectious stimuli due to its local production by different cell types, such as infiltrating immune cells, endothelial cells, and other stromal components (21). PTX3 exerts its function by binding to different ligands, including growth factors, microbial moieties, complement components, and ECM proteins (22–24). PTX3 has been reported to be upregulated and to play a protective role in various lung diseases (21). In lung infections, the protective function of PTX3 has been described in different pathological settings, including aspergillosis (25), pneumonia (26), and severe acute respiratory syndrome (27). In acute lung injury, which is strictly associated with activation of innate immune responses in the lungs, PTX3 deficiency in *Ptx3* null mice results in increased sensitivity to lung tissue damage after exposition to LPS (28). Finally, PTX3 has been proposed as a possible biomarker of disease in acute lung injury and other pulmonary disorders, including asthma, and lung carcinoma (28–30).

In the present study, we investigated the impact of endogenous PTX3 in a BLM-induced murine model of lung fibrosis. Our data show that pulmonary PTX3 expression is upregulated during lung fibrosis. In addition, by using both transgenic PTX3-overexpressing mice and *Ptx3* null animals, we demonstrate that endogenous PTX3 exerts a protective effect in BLM-induced lung fibrosis.

## Materials and Methods

### Bleomycin-Induced Pulmonary Fibrosis

Animal experiments were approved by the local animal ethics committee (OPBA, Organismo Preposto al Benessere degli Animali, Università degli Studi di Brescia, Italy) and were performed in accordance with national guidelines and regulations. Procedures involving animals and their care conformed with institutional guidelines that comply with national and international laws and policies (EEC Council Directive 86/609, OJ L 358, 12 December 1987) and with “ARRIVE” guidelines (Animals in Research Reporting *In Vivo* Experiments). Eight weeks old wild type (WT) C57BL/6, *Ptx3*^−/−^ (31) and Tie2-PTX3 (32) male mice received a single, slow intratracheal injection of 4.0 mg/kg bleomycin (B2434 Sigma-Aldrich) dissolved in 30 µl of phosphate-buffered saline (PBS). Body weight variations were monitored throughout the whole experimental period (**Supplementary Figure S1**). Mice were sacrificed at different time points (14, 21, and 28 days after treatment), and lungs collected and prepared for histopathological analysis. For each time point 5-8 mice were used for each strain (WT, Tie2-PTX3, and *Ptx3*^−/−^ animals).

### Histopathological Analysis

The left lung was fixed overnight in 10% formalin (05-01004F BioOptica), dehydrated in a graded ethanol series, embedded in paraffin and cut into 4-µm sections. Pulmonary fibrosis was analyzed using digital microscopy. For the analysis of the fibrosis area, sections were stained with Masson’s trichrome, digitalized by AperioScanScope CS Slide Scanner (Leica Biosystem, New Castle Ltd, UK) at 40x magnification and submitted to ImageScope software (Leica).

### Immunohistochemistry

Paraffin-embedded lung tissues were prepared as previously described (33). Four-µm sections were deparaffinized with xylene, incubated with 3% H_2_O_2_ in methanol for 30 min to inhibit endogenous peroxidase activity and then re-hydrated. Immunostaining was performed upon microwave or thermostat bath oven epitope retrieval in ethylene diamine tetra-acetic acid (EDTA) buffer (pH 8.00). The following primary antibodies were used: rabbit polyclonal anti-PTX3 (kind gift of B. Bottazzi, Humanitas Clinical Institute-Milan 1:100), mouse monoclonal anti-α-SMA antibody (clone 1A4, 1:300, Biocare, # CM001), rat monoclonal anti-CD45 (clone 30-F11, 1:100, BD Pharmingen, #553076), rabbit polyclonal anti-IBA1 (1:300, Wako, 019-19741), rat monoclonal anti-Ly6G (clone 1A8, 1:400, Cederlane, #AB-F118UD), mouse monoclonal anti-CD3 (clone SP7, 1:70, Leica, #565-LCE) and rabbit monoclonal PECAM-1 (clone M-20, 1:200, Santa Cruz, #SC-1506). Immunoreaction was revealed by using EnVision+ System-HRP Labelled Polymer anti-mouse or anti-rabbit (Dako) or using Rat-on-Mouse HRP-Polymer (Biocare Medical) followed by DAB as chromogen: sections were counterstained with hematoxylin. Immunostained slides were digitalized as described above and evaluated using Positive Pixel Count v9 9.0 Algorithm (Imagescope, Leica Biosystem). Staining was graded for positive pixel density (0 indicating < 25,000/mm^2^; 1, < 50,000/mm^2^; 2, <100,000/mm^2^; 3> 100,000/mm^2^ positive pixel). It must be pointed out that physiological α-SMA immuno-reactivity in perivascular and peri-bronchiolar areas was excluded in the evaluation of the samples.

### Hydroxyproline Quantification in Lungs

The right upper lung lobes were homogenized in ddH_2_O (10 mg of tissue in 100 µl). Then an aliquot was hydrolyzed in HCl 6M at 105°C for 3 h and centrifuged at 13,000 rpm for 5 min. 5 µl of the supernatant were pipetted in triplicate onto a 96 well plate and incubated at 60°C for 1 h. Collagen content was assessed using a Hydroxyproline Colorimetric Assay Kit (MAK008 Sigma-Aldrich) according to the manufacturer’s instructions.

### RNA Extraction and qPCR

Total RNA was isolated from right lower lung using TRIzol Reagent (Invitrogen) according to the manufacturer’s instructions. Two μg of total RNA were retro-transcribed with MMLV reverse transcriptase (Invitrogen) using random primers. cDNA was analyzed by quantitative real-time polymerase chain reaction (qPCR) analysis. Beta-Actin (*ActB*) was used as housekeeping gene for normalization. Primers used: Mm_*Ptx3* forward: 5′-GACCTCGGATGACTACGAG-3′, reverse: 5′-CTCCGAGTGCTCCTGGCG-3′; *Col1A1* for: 5′-TGCTCCTCTTAGGGGCCACT-3′, rev: 5′-ATTGGGGACCCTTAGGCCATT-3′; *Col1A2* for: 5′-GGTGAGCCTGGTCAAACGG-3′, rev: 5′-ACTGTGTCCTTTCACGCCTTT-3′; *Col3A1*for: 5′-CTGTAACATGGAAACTGGGGAAA-3′, rev: 5′-CCATAGCTGAACTGAAAACCACC-3′; *Acta2* for: 5′-TGCTGACAGAGGCACCACTGAA-3′, rev: 5′-CAGTTGTACGTCCAGAGGCATAG-3′; *ActB* for: 5′-CTGTCGAGTCGCGTCCACC-3′, rev: 5′-ATCGTCATCCATGGCGAACTG-3′.

### Statistical Analyses

T-test for unpaired data (2-tailed) was used to test the probability of significant differences between two groups of samples. For the statistical analysis the WT group at day 0 was used as reference. The following abbreviations were used for the indication of significance: * p<0.05; ** p<0.01; *** p<0.001.

## Results

### PTX3 Is Modulated During BLM-Induced Lung Fibrosis

**Figure 1:**
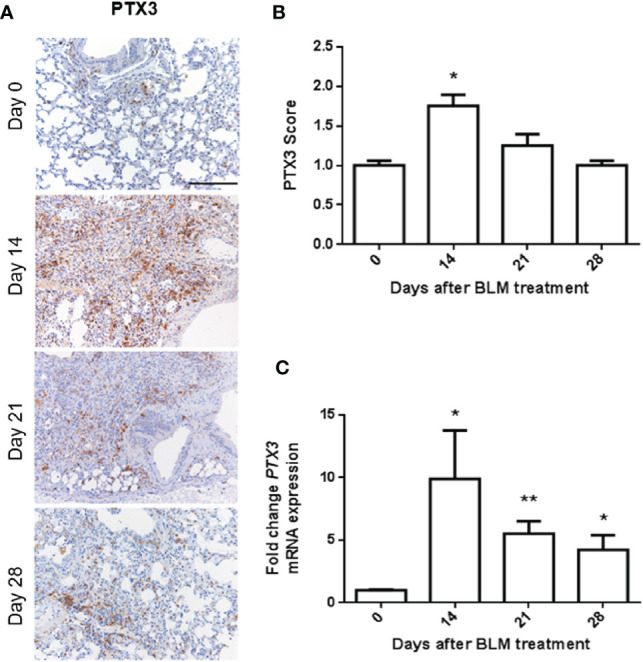
PTX3 expression is induced in BLM-induced lung fibrosis. PTX3 expression in the lungs of C57BL/6 wild type mice treated with bleomycin (BLM) and sacrificed at day 0, 14, 21, and 28 after treatment was detected by immunohistochemistry **(A)** and quantified by scoring the immunostaining **(B)** or by qPCR **(C)**. *N* = 5–8 mice/group; scale bar = 200 µm; *P < 0.05, **p < 0.01.

Intratracheal administration of BLM induces peri-bronchial lung fibrosis in mice, detectable 14 days after treatment and progressing up to day 21, to decrease at/after day 28 (15). On this basis, in order to evaluate the modulation of PTX3 expression in the lungs during fibrosis onset, C57BL/6 male mice were treated intratracheal with BLM and sacrificed after 14, 21, and 28 days. Then, harvested lungs were processed for immunohistochemical (IHC) and qPCR analyses. In keeping with its role as an “early” response to damaging and inflammatory stimuli (21), PTX3 immuno-reactivity was significantly increased in fibrotic lung sections 14 days after BLM treatment, to decrease to basal levels at day 28 (**Figures 1A, B**). These data were confirmed by qPCR analysis that demonstrated a significant upregulation of *Ptx3* expression at day 14 and its gradual decrease thereafter (**Figure 1C**).

### PTX3 Exerts a Protective Role in Lung Fibrosis

**Figure 2:**
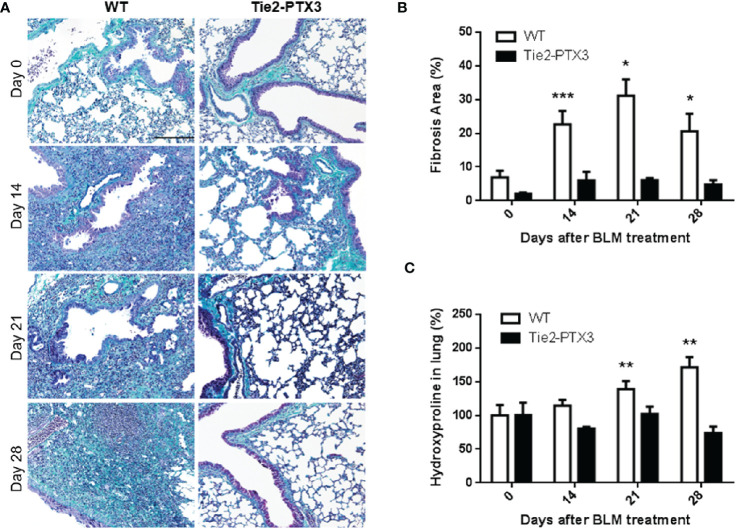
Stromal PTX3 overexpression prevents lung fibrosis in BLM-treated mice. Fibrosis in lungs of wild type (WT) and transgenic Tie2-PTX3 mice treated with bleomycin (BLM) was determined by Masson’s trichrome staining **(A)**, subsequent quantification of fibrotic area **(B)**, and quantification of hydroxyproline in lungs extracts **(C)**. *N* = 5–8 mice/group; scale bar = 200 µm; *P < 0.05, **p < 0.01, ***p < 0.001.

Previous observations had shown a potential role of PTX3 as a protective factor in different pathological settings in the lungs (21). On this basis, we evaluated the impact of PTX3 overexpression during the onset and progression of the fibrotic process in the lungs by taking advantage of a transgenic Tie2-PTX3 murine model. In this model the overexpression of the human *PTX3* gene (**Supplementary Figure S2**) is driven by the endothelial-specific *Tie2* promoter, leading to the stromal accumulation of high levels of the PTX3 protein in all tissues, including the lungs, without any significant impact on normal development of organs and tissues (32, 34). Wild type (WT) and transgenic Tie2-PTX3 mice were treated intratracheal with BLM and the progression of lung fibrosis was investigated. As shown in **Figures 2A, B**, quantification of fibrotic/collagen positive areas after trichrome staining of lung sections revealed an increase of fibrotic tissue in WT animals from day 14 to day 21, with a partial regression at day 28. When compared to WT mice, fibrotic areas were significantly reduced in Tie2-PTX3 mice at all the time points investigated, close to the levels measured in untreated animals. Accordingly, the levels of hydroxyproline, a marker for the presence of collagen in the ECM, were significantly higher in WT lungs when compared with those measured in Tie2-PTX3 animals (**Figure 2C**).

**Figure 3:**
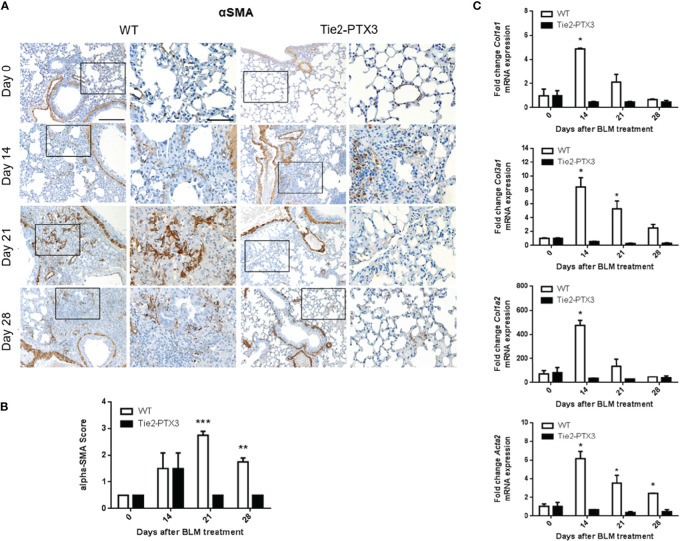
Stromal PTX3 overexpression reduces fibroblast activation in BLM-induced lung fibrosis. **(A)** Immunohistochemical analysis of αSMA expression in the lungs of wild type (WT) and transgenic Tie2-PTX3 mice treated with bleomycin (BLM). For each image, the boxed area is shown at higher magnification in the right panel. Scale bar = 100 µm; scale bar of magnified images = 200 µm. **(B)** Score quantification of the αSMA immunostaining. αSMA immuno-reactivity in perivascular and peri-bronchiolar areas was excluded from the quantification. **(C)** qPCR analysis on lung extracts for the expression of collagen subunits and *Acta2* mRNA. *N* = 5–8 mice/group; *P < 0.05, **p < 0.01, ***p < 0.001.

Further characterization of BLM-treated lungs showed a significant increase of areas with alpha-smooth muscle actin (αSMA^+^) positive fibroblasts in WT mice when compared with Tie2-PTX3 animals (**Figures 3A, B**). Related to this fibroblastic reaction, upregulation of the expression of *Col1a1*, *Col1a2*, and *Col3a1* collagen subunits and of the αSMA-encoding *Acta2* gene was detected by qPCR in the lungs of WT but not of Tie2-PTX3 mice (**Figure 3C**). In addition, foci of angiogenesis, represented by CD31^+^ endothelial cells, were detectable in the fibrotic areas of BLM-treated mice with no apparent difference between WT and Tie2-PTX3 groups (**Supplementary Figure S3**).

**Figure 4:**
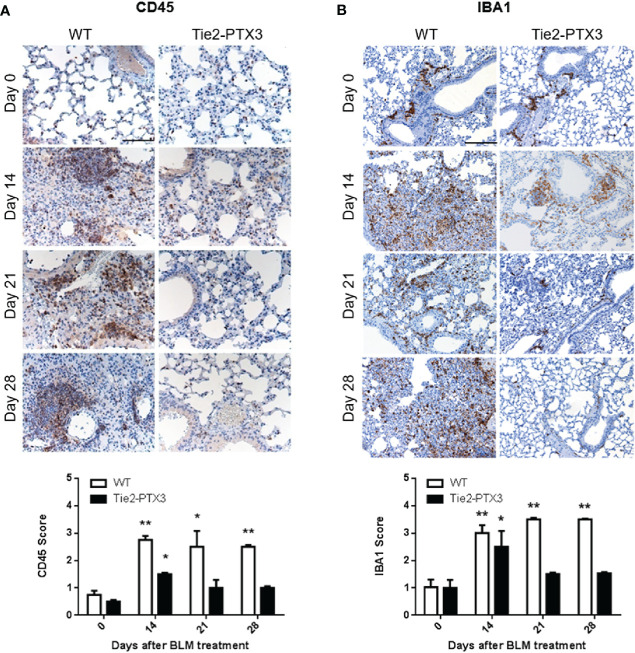
Stromal PTX3 overexpression prevents immune infiltration in BLM-induced lung fibrosis. CD45 **(A)** and IBA1 **(B)** levels in the lungs of wild type (WT) and transgenic Tie2-PTX3 mice treated with bleomycin (BLM) were assessed by immunohistochemistry and quantified by scoring the immunostaining. *N* = 5–8 mice/group; scale bar = 100 µm **(A)**, 200 µm **(B)**; *P < 0.05; **p < 0.01.

The inflammatory infiltrate plays a pivotal role during the response to tissue damage that drives fibrosis (7, 8). In order to quantify the overall immune infiltrate in BLM-treated lungs, CD45 immunostaining was performed on samples from the different groups. As shown in **Figure 4A**, a vast CD45^+^ cell infiltrate was detected in WT lungs, while a significant reduction of CD45^+^ cells was observed in Tie2-PTX3 lungs at all the time points investigated. These observations are in line with the limited fibrotic response occurring in Tie2-PTX3 animals. Accordingly, in keeping with the pivotal role played by macrophages in BLM-induced fibrosis (7), IBA1^+^ macrophages were widely present in fibrotic WT lungs throughout the whole experimental period and consistently reduced in Tie2-PTX3 lungs that showed a detectable macrophagic infiltrate only ad day 14 (**Figure 4B**). In addition, a further characterization of the immune infiltrate at the time points considered demonstrate that CD3^+^ lymphocytes increase in both WT and Tie2-PTX3 at day 14 after treatment, to decrease more rapidly in PTX3 overexpressing animals in respect to controls (**Supplementary Figure S4A**). Finally, very few Ly6G^+^ neutrophils were present in the lungs of treated mice, with no significant differences between the WT and Tie2-PTX3 animals (**Supplementary Figure S4B**).

**Figure 5:**
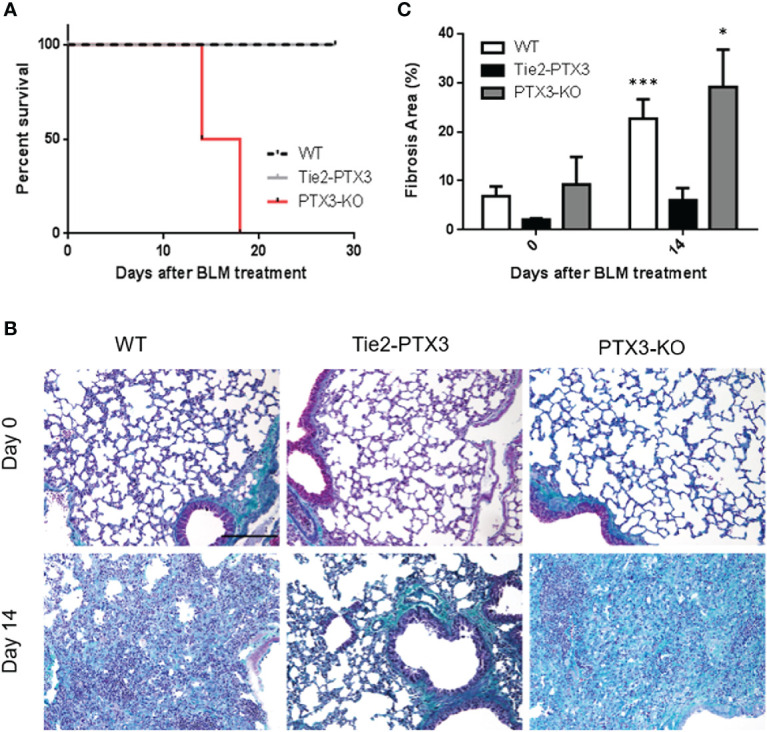
Ptx3 knockdown increases the deposition of fibrotic tissue and a rapid animal death in BLM-induced lung fibrosis. **(A)** Kaplan-Meier survival curve of wild type (WT) and transgenic Tie2-PTX3 and *Ptx3*^−/−^ (KO) mice treated with bleomycin (BLM). Masson’s trichrome staining **(B)** and quantification of the fibrotic area **(C)** performed on lungs at day 0 and day 14 after treatment. *N* = 5–8 mice/group; scale bar = 100 µm; *P <0 .05, ***p < 0.001.

Together, these data indicate that PTX3 overexpression exerts a protective impact on BLM-induced fibrosis in transgenic Tie2-PTX3 mice. These findings, together with the observation that a significant upregulation of PTX3 expression occurs in WT animals during BLM-induced fibrosis (see above), prompted us to assess the role, if any, of endogenous PTX3 on this process. To this aim, we took advantage from genetically deficient *Ptx3*^−/−^ mice that lack endogenous PTX3 expression in all organs, including lungs, and are characterized by a normal pre and post-natal development with no signs of inherited pathological conditions with the exception of subfertility in female animals (31). On the other hand, these mice may show different responses under defined experimental conditions characterized by the involvement of the innate immune arm [reviewed in (23, 35, 36)]. BLM was administered intratracheal to *Ptx3* null mice and lung fibrosis was followed in parallel to that occurring in WT and Tie2-PTX3 animals. As shown in **Figure 5A**, BLM treatment caused a dramatic decrease of the lifespan of *Ptx3*^−/−^ mice, with 4 out of 8 mice dying at day 14 and no survival occurring at day 21after treatment. At variance, no animal death was observed for BLM-treated WT and Tie2-PTX3 mice. Accordingly, trichrome staining of the lung sections and quantification of fibrotic areas confirmed a significant increase of fibrotic/scar tissue at day 14 in PTX3^−/−^ mice when compared to WT and Tie2-PTX3 animals (**Figures 5B, C**).

## Discussion

Hyper-proliferating fibroblasts/myofibroblasts and augmented deposition of ECM are typical features of IPF and the main cause of lung architecture alterations underlying the loss of respiratory function (6). This is paralleled by an inflammatory response whose contribution to IPF has long been debated (37) and in some cases remains controversial (38, 39). For instance, treatment of IPF patients with steroids reduces inflammation in the lung but does not improve survival nor clinical outcome (40, 41), and a clinical trial based on immune-suppression was discontinued due to increased hospitalization and death (42).

PTX3 is a component of the humoral arm of the innate immunity. Even though its expression in human tissues is usually associated with inflammation, both pro-inflammatory and inflammation-limiting properties have been reported in preclinical models of disease, and a possible dual role has been unveiled in physiological and pathological settings (35, 43, 44). On one side, PTX3 generally exhibits protective antibody-like functions and promotes tissue repair *via* matrix remodeling and modulation of the inflammatory response. On the other hand, potential damaging effects have been ascribed to PTX3 due to its capacity to induce endothelial dysfunction (45), exacerbated complement activation and inflammation (44, 46, 47).

In inflammatory/injury settings, neutrophils are among the first leukocytes to be recruited. Once activated, they secrete their content of “ready to release” PTX3 that partially remains associated with neutrophil extracellular traps (NETs) (48, 49). On the other hand, PTX3 locally released by activated leukocytes may impair leukocyte rolling on endothelium, thus attenuating the recruitment of neutrophils, regulating inflammation, and reducing tissue damage in murine models of acute lung injury, pleurisy, and mesenteric inflammation (50).

Under physiological conditions, the expression levels of PTX3 are low in the lungs of healthy mice and can be modulated depending on the severity and duration of the damaging agent (31, 51–53). In this study we investigated the expression of PTX3 in a BLM-induced murine model of lung fibrosis with the aim to define its role in this pathological context.

In keeping with previous observations in preclinical models of lung fibrosis and in lung tissue from IPF patients (54), PTX3 expression rapidly increases in the lungs of BLM-treated animals both at mRNA and protein levels. This upregulation is transient, reaches a peak at 14 days after treatment to decrease to physiological levels thereafter. Thus, PTX3 upregulation represents an early event in response to the fibrotic insult, unable to restrain and control the onset of BLM-induced fibrosis in the lungs, that continues till day 28 after treatment.

Two distinct experimental approaches pointed to a protective role for PTX3 in this model of PF. In a first set of experiments, PTX3 overexpression in transgenic Tie2-PTX3 animals, which results in the accumulation of PTX3 in the lung stroma (32), was able to limit the formation of fibrotic tissue in the lungs, with reduced activation of fibroblasts and ECM deposition. Moreover, the infiltration of immune CD45^+^ cells was reduced in Tie2-PTX3 lungs that showed a dramatic decrease in infiltrating IBA1^+^ macrophages when compared to WT animals, with moderate or no major differences in infiltrating neutrophils and T lymphocytes at the experimental points investigated. These observations are in line with the critical regulatory activities exerted by macrophages during all the steps of fibrosis onset and repair (55, 56). The protective effect of PTX3 on lung fibrosis was confirmed in a second set of experiments performed on *Ptx3*^−/−^ animals in which PTX3 deficiency caused an increased deposition of fibrotic tissue and a rapid animal death following BLM administration.

Even though the molecular mechanisms at the basis of this impact of PTX3 on PF remain to be elucidated, previous experimental evidences suggest that its accumulation in the pulmonary stroma may modulate different biological processes, including the recruitment of immune cells (50, 57, 58) and fibroblast activation (22, 36). This occurs *via* the interaction of PTX3 with growth factors, complement components, the haemostatic system and the fibrinolytic cascade. Indeed, PTX3 can bind to fibroblast growth factors (FGFs) and impair the FGF/FGF receptor system (22, 24) which plays a relevant role in fibrosis (59) as well as in immune cell recruitment (33, 60). *In vitro*, the interaction of PTX3 with FcγRI has been shown to favor the differentiation of human and murine monocytes into fibrocytes, fibroblast-like cells expressing both haematopoietic and stromal cell markers frequently found in fibrotic lesions (54, 61). In the same context, the serum amyloid P component (SAP) was shown to play a fibrocyte-inhibitory activity stronger than PTX3 (54), and injections of SAP inhibited fibrosis in various mouse models of the disease (62, 63). Interestingly, SAP expression was almost absent in fibrotic areas, while PTX3 was widespread, thus suggesting that relative levels of SAP and PTX3 may have a significant role in fibrocyte differentiation at fibrotic sites (54).

In the acute lung injury, a strong activation of the innate immune system in the lungs is accompanied by the expression of PTX3. This pathological context is characterized by infiltration of neutrophils and increased production of nitric oxide and of tissue factor. It has been suggested that high levels of PTX3 activate the local innate immune system and play a protective role against lung insults (21, 51). Accordingly, *Ptx3*^−/−^ mice are more susceptible to tissue damage after exposure to LPS (28). In addition, PTX3 can interact with fibrin and plasminogen under acidic conditions in damaged/inflamed tissues, fostering the remodeling of the fibrin-rich matrix and tissue repair (64).

Overall, in this study we have reported the observational results of the effect of endogenous PTX3 modulation on lung fibrosis onset and phenotype. Intravenous or intraperitoneal treatment with recombinant PTX3 has resulted in beneficial outcomes in various experimental models of inflammatory diseases (25, 31, 48, 65–67). Thus, in a therapeutic perspective, it will be of importance to assess the effect of recombinant PTX3 to prevent fibrosis in BLM-treated mice. Our results and these future studies will pave the way for the characterization of the molecular players modulated by PTX3 in PF and for the design of PTX3-derived approaches to be used for the treatment of PF patients.

## Data Availability Statement

The raw data supporting the conclusions of this article will be made available by the authors, without undue reservation.

## Ethics Statement

The animal study was reviewed and approved by OPBA University of Brescia.

## Author Contributions

FM performed *in vivo* experiments. MB performed immunohistochemistry. AS, WV, and SG gave technical assistance. MP conceived the experimental plan and revised the paper. RR conceived and supervised the experiments, and wrote the paper. All authors contributed to the article and approved the submitted version.

## Funding

This work was supported by Associazione Italiana per la Ricerca sul Cancro (IG 2019 no. 23116 to MP and IG 2019 no. 23151 to RR). FM was supported by Fondazione Veronesi Fellowship.

## Conflict of Interest

The authors declare that the research was conducted in the absence of any commercial or financial relationships that could be construed as a potential conflict of interest.

## References

[1] Bartczak K Bialas AJ Kotecki MJ Gorski P Piotrowski WJ. More than a Genetic Code: Epigenetics of Lung Fibrosis. Mol Diagn Ther (2020) 24(6):665–81. 10.1007/s40291-020-00490-7 PMC7677145 32926347

[2] Sauleda J Nunez B Sala E Soriano JB. Idiopathic Pulmonary Fibrosis: Epidemiology, Natural History, Phenotypes. Med Sci (Basel) (2018) 6(4). 10.3390/medsci6040110 PMC6313500 30501130

[3] Diridollou T Sohier L Rousseau C Angibaud A Chauvin P Gaignon T. Idiopathic pulmonary fibrosis: Significance of the usual interstitial pneumonia (UIP) CT-scan patterns defined in new international guidelines. Respir Med Res (2020) 77:72–8. 10.1016/j.resmer.2020.02.004 32416587

[4] Raghu G Collard HR Egan JJ Martinez FJ Behr J Brown KK. An official ATS/ERS/JRS/ALAT statement: idiopathic pulmonary fibrosis: evidence-based guidelines for diagnosis and management. Am J Respir Crit Care Med (2011) 183(6):788–824. 10.1164/rccm.2009-040GL 21471066 PMC5450933

[5] Lederer DJ Martinez FJ. Idiopathic Pulmonary Fibrosis. N Engl J Med (2018) 379(8):797–8. 10.1056/NEJMc1807508 30134133

[6] Kuhn C Mason RJ. Immunolocalization of SPARC, tenascin, and thrombospondin in pulmonary fibrosis. Am J Pathol (1995) 147(6):1759–69. PMC1869942 7495300

[7] Laskin DL Malaviya R Laskin JD. Role of Macrophages in Acute Lung Injury and Chronic Fibrosis Induced by Pulmonary Toxicants. Toxicol Sci (2019) 168(2):287–301. 10.1093/toxsci/kfy309 30590802 PMC6432864

[8] Misharin AV Morales-Nebreda L Reyfman PA Cuda CM Walter JM McQuattie-Pimentel AC. Monocyte-derived alveolar macrophages drive lung fibrosis and persist in the lung over the life span. J Exp Med (2017) 214(8):2387–404. 10.1084/jem.20162152 PMC5551573 28694385

[9] Larson-Casey JL Deshane JS Ryan AJ Thannickal VJ Carter AB. Macrophage Akt1 Kinase-Mediated Mitophagy Modulates Apoptosis Resistance and Pulmonary Fibrosis. Immunity (2016) 44(3):582–96. 10.1016/j.immuni.2016.01.001 PMC4794358 26921108

[10] Gibbons MA MacKinnon AC Ramachandran P Dhaliwal K Duffin R Phythian-Adams AT. Ly6Chi monocytes direct alternatively activated profibrotic macrophage regulation of lung fibrosis. Am J Respir Crit Care Med (2011) 184(5):569–81. 10.1164/rccm.201010-1719OC 21680953

[11] Bickelhaupt S Erbel C Timke C Wirkner U Dadrich M Flechsig P. Effects of CTGF Blockade on Attenuation and Reversal of Radiation-Induced Pulmonary Fibrosis. J Natl Cancer Inst (2017) 109(8). 10.1093/jnci/djw339 28376190

[12] Prasse A Pechkovsky DV Toews GB Jungraithmayr W Kollert F Goldmann T. A vicious circle of alveolar macrophages and fibroblasts perpetuates pulmonary fibrosis via CCL18. Am J Respir Crit Care Med (2006) 173(7):781–92. 10.1164/rccm.200509-1518OC 16415274

[13] Pulichino AM Wang IM Caron A Mortimer J Auger A Boie Y. Identification of transforming growth factor beta1-driven genetic programs of acute lung fibrosis. Am J Respir Cell Mol Biol (2008) 39(3):324–36. 10.1165/rcmb.2007-0186OC 18403781

[14] Shvedova AA Kisin ER Mercer R Murray AR Johnson VJ Potapovich AI. Unusual inflammatory and fibrogenic pulmonary responses to single-walled carbon nanotubes in mice. Am J Physiol Lung Cell Mol Physiol (2005) 289(5):L698–708. 10.1152/ajplung.00084.2005 15951334

[15] Moore BB Hogaboam CM. Murine models of pulmonary fibrosis. Am J Physiol Lung Cell Mol Physiol (2008) 294(2):L152–60. 10.1152/ajplung.00313.2007 17993587

[16] Izbicki G Segel MJ Christensen TG Conner MW Breuer R. Time course of bleomycin-induced lung fibrosis. Int J Exp Pathol (2002) 83(3):111–9. 10.1046/j.1365-2613.2002.00220.x PMC2517673 12383190

[17] Muggia FM Louie AC Sikic BI. Pulmonary toxicity of antitumor agents. Cancer Treat Rev (1983) 10(4):221–43. 10.1016/0305-7372(83)90012-9 6198083

[18] Phan SH Thrall RS Ward PA. Bleomycin-induced pulmonary fibrosis in rats: biochemical demonstration of increased rate of collagen synthesis. Am Rev Respir Dis (1980) 121(3):501–6. 10.1164/arrd.1980.121.3.501 6158281

[19] Snider GL Hayes JA Korthy AL. Chronic interstitial pulmonary fibrosis produced in hamsters by endotracheal bleomycin: pathology and stereology. Am Rev Respir Dis (1978) 117(6):1099–108. 10.1164/arrd.1978.117.6.1099 78675

[20] Thrall RS McCormick JR Jack RM McReynolds RA Ward PA. Bleomycin-induced pulmonary fibrosis in the rat: inhibition by indomethacin. Am J Pathol (1979) 95(1):117–30. PMC2042298 86304

[21] Balhara J Koussih L Zhang J Gounni AS. Pentraxin 3: an immuno-regulator in the lungs. Front Immunol (2013) 4:127. 10.3389/fimmu.2013.00127 23755050 PMC3668324

[22] Giacomini A Ghedini GC Presta M Ronca R. Long pentraxin 3: A novel multifaceted player in cancer. Biochim Biophys Acta (2018) 1869(1):53–63. 10.1016/j.bbcan.2017.11.004 29175552

[23] Doni A Stravalaci M Inforzato A Magrini E Mantovani A Garlanda C. The Long Pentraxin PTX3 as a Link Between Innate Immunity, Tissue Remodeling, and Cancer. Front Immunol (2019) 10:712. 10.3389/fimmu.2019.00712 31019517 PMC6459138

[24] Presta M Foglio E Churruca Schuind A Ronca R. Long Pentraxin-3 Modulates the Angiogenic Activity of Fibroblast Growth Factor-2. Front Immunol (2018) 9:2327. 10.3389/fimmu.2018.02327 30349543 PMC6187966

[25] Moalli F Doni A Deban L Zelante T Zagarella S Bottazzi B. Role of complement and Fc{gamma} receptors in the protective activity of the long pentraxin PTX3 against Aspergillus fumigatus. Blood (2010) 116(24):5170–80. 10.1182/blood-2009-12-258376 20829368

[26] Deban L Jarva H Lehtinen MJ Bottazzi B Bastone A Doni A. Binding of the long pentraxin PTX3 to factor H: interacting domains and function in the regulation of complement activation. J Immunol (2008) 181(12):8433–40. 10.4049/jimmunol.181.12.8433 19050261

[27] Han B Ma X Zhang J Zhang Y Bai X Hwang DM. Protective effects of long pentraxin PTX3 on lung injury in a severe acute respiratory syndrome model in mice. Lab Investigation J Tech Methods Pathol (2012) 92(9):1285–96. 10.1038/labinvest.2012.92 PMC3955193 22732935

[28] Okutani D Han B Mura M Waddell TK Keshavjee S Liu M. High-volume ventilation induces pentraxin 3 expression in multiple acute lung injury models in rats. Am J Physiol Lung Cell Mol Physiol (2007) 292(1):L144–53. 10.1152/ajplung.00002.2006 16936248

[29] Zhang J Shan L Koussih L Redhu NS Halayko AJ Chakir J. Pentraxin 3 (PTX3) expression in allergic asthmatic airways: role in airway smooth muscle migration and chemokine production. PLoS One (2012) 7(4):e34965. 10.1371/journal.pone.0034965 22529962 PMC3329534

[30] Diamandis EP Goodglick L Planque C Thornquist MD. Pentraxin-3 is a novel biomarker of lung carcinoma. Clin Cancer Res (2011) 17(8):2395–9. 10.1158/1078-0432.CCR-10-3024 21257721

[31] Garlanda C Hirsch E Bozza S Salustri A De Acetis M Nota R. Non-redundant role of the long pentraxin PTX3 in anti-fungal innate immune response. Nature (2002) 420(6912):182–6. 10.1038/nature01195 12432394

[32] Ronca R Giacomini A Di Salle E Coltrini D Pagano K Ragona L. Long-Pentraxin 3 Derivative as a Small-Molecule FGF Trap for Cancer Therapy. Cancer Cell (2015) 28(2):225–39. 10.1016/j.ccell.2015.07.002 26267536

[33] Ronca R Tamma R Coltrini D Ruggieri S Presta M Ribatti D. Fibroblast growth factor modulates mast cell recruitment in a murine model of prostate cancer. Oncotarget (2017) 8(47):82583–92. 10.18632/oncotarget.19773 PMC5669912 29137286

[34] Matarazzo S Melocchi L Rezzola S Grillo E Maccarinelli F Giacomini A. Long Pentraxin-3 Follows and Modulates Bladder Cancer Progression. Cancers (2019) 11(9). 10.3390/cancers11091277 PMC6770810 31480336

[35] Mantovani A Valentino S Gentile S Inforzato A Bottazzi B Garlanda C. The long pentraxin PTX3: a paradigm for humoral pattern recognition molecules. Ann N Y Acad Sci (2013) 1285:1–14. 10.1111/nyas.12043 23527487

[36] Doni A Garlanda C Mantovani A. Innate immunity, hemostasis and matrix remodeling: PTX3 as a link. Semin Immunol (2016) 28(6):570–7. 10.1016/j.smim.2016.10.012 PMC5414833 27881292

[37] McLean-Tooke A Moore I Lake F. Idiopathic and immune-related pulmonary fibrosis: diagnostic and therapeutic challenges. Clin Trans Immunol (2019) 8(11):e1086. 10.1002/cti2.1086 PMC6831929 31709050

[38] Gauldie J. Pro: Inflammatory mechanisms are a minor component of the pathogenesis of idiopathic pulmonary fibrosis. Am J Respir Crit Care Med (2002) 165(9):1205–6. 10.1164/rccm.2202054 11991866

[39] Strieter RM. Con: Inflammatory mechanisms are not a minor component of the pathogenesis of idiopathic pulmonary fibrosis. Am J Respir Crit Care Med (2002) 165(9):1206–7; discussion 7-8. 10.1164/rccm.2202055 11991867

[40] Grijm K Verberne HJ Krouwels FH Weller FR Jansen HM Bresser P. Semiquantitative 67Ga scintigraphy as an indicator of response to and prognosis after corticosteroid treatment in idiopathic interstitial pneumonia. J Nucl Med (2005) 46(9):1421–6. 16157523

[41] Pereira CA Malheiros T Coletta EM Ferreira RG Rubin AS Otta JS. Survival in idiopathic pulmonary fibrosis-cytotoxic agents compared to corticosteroids. Respir Med (2006) 100(2):340–7. 10.1016/j.rmed.2005.05.008 16002271

[42] Raghu G Anstrom KJ King TE Jr. Lasky JA Martinez FJ. Prednisone, azathioprine, and N-acetylcysteine for pulmonary fibrosis. N Engl J Med (2012) 366(21):1968–77. 10.1056/NEJMoa1113354 PMC3422642 22607134

[43] Bonacina F Baragetti A Catapano AL Norata GD. Long pentraxin 3: experimental and clinical relevance in cardiovascular diseases. Mediators Inflamm (2013) 2013:725102. 10.1155/2013/725102 23690668 PMC3649691

[44] Magrini E Mantovani A Garlanda C. The Dual Complexity of PTX3 in Health and Disease: A Balancing Act? Trends Mol Med (2016) 22(6):497–510. 10.1016/j.molmed.2016.04.007 27179743 PMC5414840

[45] Foo SS Chen W Taylor A Sheng KC Yu X Teng TS. Role of pentraxin 3 in shaping arthritogenic alphaviral disease: from enhanced viral replication to immunomodulation. PLoS Pathog (2015) 11(2):e1004649. 10.1371/journal.ppat.1004649 25695775 PMC4335073

[46] Inforzato A Doni A Barajon I Leone R Garlanda C Bottazzi B. PTX3 as a paradigm for the interaction of pentraxins with the complement system. Semin Immunol (2013) 25(1):79–85. 10.1016/j.smim.2013.05.002 23747040

[47] Souza DG Soares AC Pinho V Torloni H Reis LF Teixeira MM. Increased mortality and inflammation in tumor necrosis factor-stimulated gene-14 transgenic mice after ischemia and reperfusion injury. Am J Pathol (2002) 160(5):1755–65. 10.1016/s0002-9440(10)61122-4 PMC1850862 12000727

[48] Jaillon S Peri G Delneste Y Fremaux I Doni A Moalli F. The humoral pattern recognition receptor PTX3 is stored in neutrophil granules and localizes in extracellular traps. J Exp Med (2007) 204(4):793–804. 10.1084/jem.20061301 17389238 PMC2118544

[49] Daigo K Yamaguchi N Kawamura T Matsubara K Jiang S Ohashi R. The proteomic profile of circulating pentraxin 3 (PTX3) complex in sepsis demonstrates the interaction with azurocidin 1 and other components of neutrophil extracellular traps. Mol Cell Proteomics MCP (2012) 11(6):M111 015073. 10.1074/mcp.M111.015073 PMC3433926 22278372

[50] Deban L Russo RC Sironi M Moalli F Scanziani M Zambelli V. Regulation of leukocyte recruitment by the long pentraxin PTX3. Nat Immunol (2010) 11(4):328–34. 10.1038/ni.1854 20208538

[51] He X Han B Liu M. Long pentraxin 3 in pulmonary infection and acute lung injury. Am J Physiol Lung Cell Mol Physiol (2007) 292(5):L1039–49. 10.1152/ajplung.00490.2006 17277044

[52] Pauwels NS Bracke KR Maes T Van Pottelberge GR Garlanda C Mantovani A. Cigarette smoke induces PTX3 expression in pulmonary veins of mice in an IL-1 dependent manner. Respir Res (2010) 11:134. 10.1186/1465-9921-11-134 20920344 PMC2959025

[53] Han B Mura M Andrade CF Okutani D Lodyga M dos Santos CC. TNFalpha-induced long pentraxin PTX3 expression in human lung epithelial cells via JNK. J Immunol (2005) 175(12):8303–11. 10.4049/jimmunol.175.12.8303 16339571

[54] Pilling D Cox N Vakil V Verbeek JS Gomer RH. The long pentraxin PTX3 promotes fibrocyte differentiation. PLoS One (2015) 10(3):e0119709. 10.1371/journal.pone.0119709 25774777 PMC4361553

[55] Wynn TA Barron L. Macrophages: master regulators of inflammation and fibrosis. Semin Liver Dis (2010) 30(3):245–57. 10.1055/s-0030-1255354 PMC2924662 20665377

[56] Wynn TA Vannella KM. Macrophages in Tissue Repair, Regeneration, and Fibrosis. Immunity (2016) 44(3):450–62. 10.1016/j.immuni.2016.02.015 PMC4794754 26982353

[57] Bonavita E Gentile S Rubino M Maina V Papait R Kunderfranco P. PTX3 is an extrinsic oncosuppressor regulating complement-dependent inflammation in cancer. Cell (2015) 160(4):700–14. 10.1016/j.cell.2015.01.004 25679762

[58] Annese T Ronca R Tamma R Giacomini A Ruggieri S Grillo E. PTX3 Modulates Neovascularization and Immune Inflammatory Infiltrate in a Murine Model of Fibrosarcoma. Int J Mol Sci (2019) 20(18). 10.3390/ijms20184599 PMC6770794 31533326

[59] Guzy RD Li L Smith C Dorry SJ Koo HY Chen L. Pulmonary fibrosis requires cell-autonomous mesenchymal fibroblast growth factor (FGF) signaling. J Biol Chem (2017) 292(25):10364–78. 10.1074/jbc.M117.791764 PMC5481550 28487375

[60] Presta M Andres G Leali D Dell’Era P Ronca R. Inflammatory cells and chemokines sustain FGF2-induced angiogenesis. Eur Cytokine Netw (2009) 20(2):39–50. 10.1684/ecn.2009.0155 19541589

[61] Reilkoff RA Bucala R Herzog EL. Fibrocytes: emerging effector cells in chronic inflammation. Nat Rev Immunol (2011) 11(6):427–35. 10.1038/nri2990 PMC3599774 21597472

[62] Pilling D Roife D Wang M Ronkainen SD Crawford JR Travis EL. Reduction of bleomycin-induced pulmonary fibrosis by serum amyloid P. J Immunol (2007) 179(6):4035–44. 10.4049/jimmunol.179.6.4035 PMC4482349 17785842

[63] Murray LA Rosada R Moreira AP Joshi A Kramer MS Hesson DP. Serum amyloid P therapeutically attenuates murine bleomycin-induced pulmonary fibrosis via its effects on macrophages. PLoS One (2010) 5(3):e9683. 10.1371/journal.pone.0009683 20300636 PMC2837381

[64] Doni A Musso T Morone D Bastone A Zambelli V Sironi M. An acidic microenvironment sets the humoral pattern recognition molecule PTX3 in a tissue repair mode. J Exp Med (2015) 212(6):905–25. 10.1084/jem.20141268 PMC4451130 25964372

[65] Gaziano R Bozza S Bellocchio S Perruccio K Montagnoli C Pitzurra L. Anti-Aspergillus fumigatus efficacy of pentraxin 3 alone and in combination with antifungals. Antimicrobial Agents Chemother (2004) 48(11):4414–21. 10.1128/AAC.48.11.4414-4421.2004 PMC525434 15504871

[66] Paroni M Moalli F Nebuloni M Pasqualini F Bonfield T Nonis A. Response of CFTR-deficient mice to long-term chronic Pseudomonas aeruginosa infection and PTX3 therapy. J Infect Dis (2013) 208(1):130–8. 10.1093/infdis/jis636 PMC4055833 23087427

[67] Moalli F Paroni M Veliz Rodriguez T Riva F Polentarutti N Bottazzi B. The therapeutic potential of the humoral pattern recognition molecule PTX3 in chronic lung infection caused by Pseudomonas aeruginosa. J Immunol (2011) 186(9):5425–34. 10.4049/jimmunol.1002035 21441447

