# Supplementary material for: Endogenous Long Pentraxin 3 Exerts a Protective Role in a Murine Model of Pulmonary Fibrosis
Source: Front Immunol. 2021 Feb 18;12:617671. doi: 10.3389/fimmu.2021.617671 (PMC7930377; doi:10.3389/fimmu.2021.617671)
Supplement: Supplementary file 1 [file DataSheet_1.pdf]

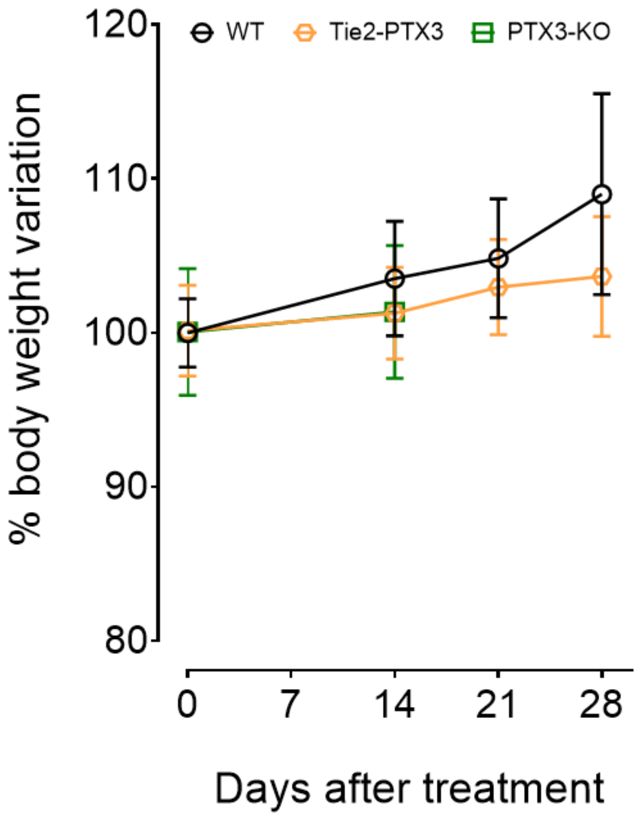

Supplementary Figure S1. Body weight variations of BLM-treated animals is shown as percentage (%) in respect to the initial body weight. Data are the mean ± SEM; *N* = 5-8 mice/group.

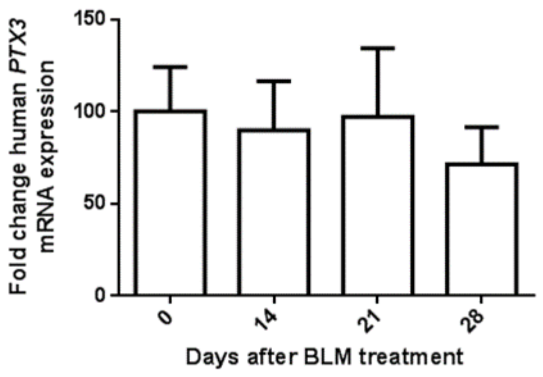

Supplementary Figure S2. qPCR analysis of human *PTX3* expression in transgenic Tie2-PTX3 animals during BLM treatment. Data are the mean ± SEM; *N* = 5-8 mice/group.

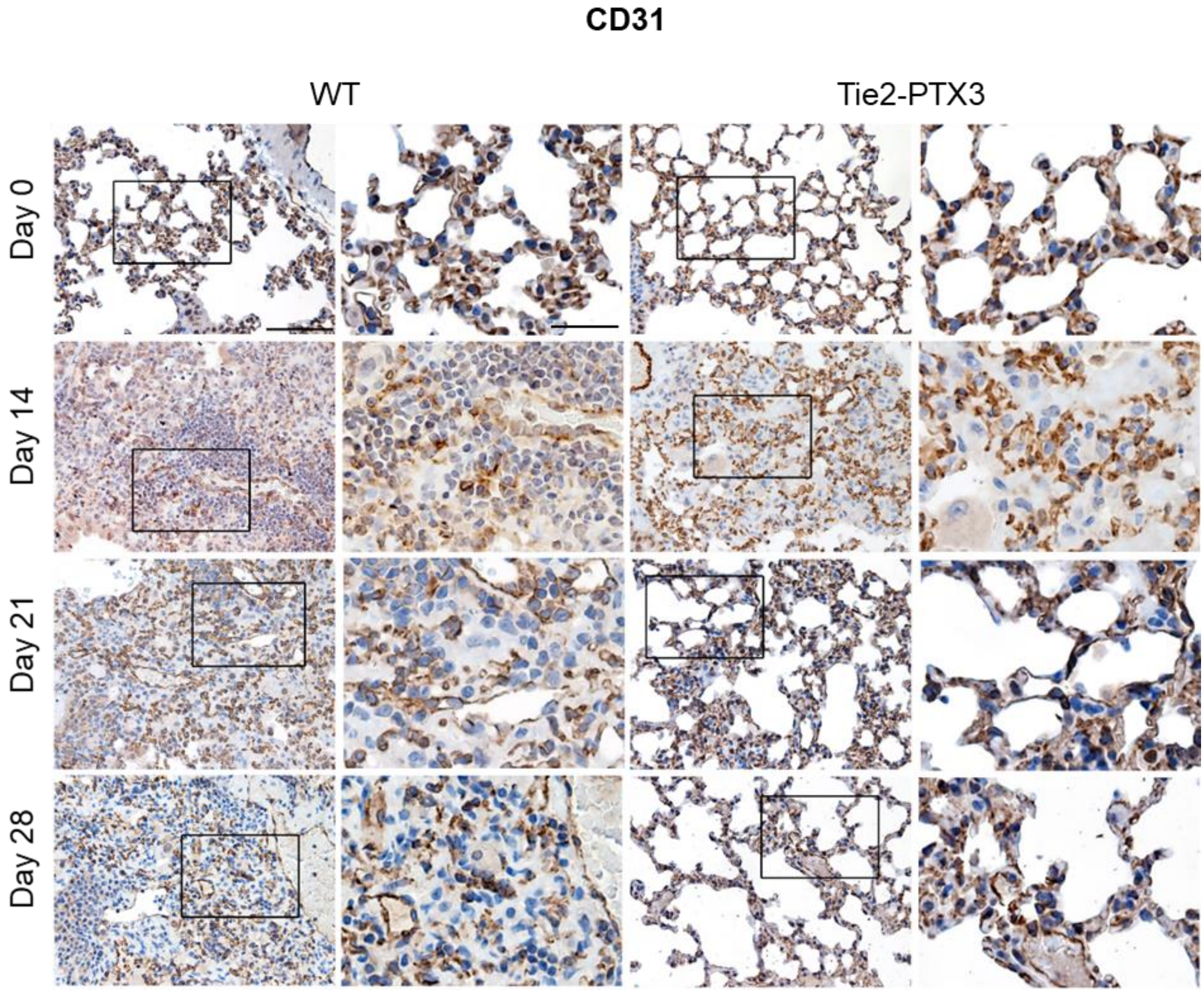

Supplementary Figure S3. Representative images of the immunostaining for CD31 performed on the lungs of BLM-treated WT and Tie2-PTX3 animals. For each image, the boxed area is shown at higher magnification in the right panel. *N* = 5-8 mice/group; scale bar = 100 µm; scale bar for magnified pictures = 200 µm.

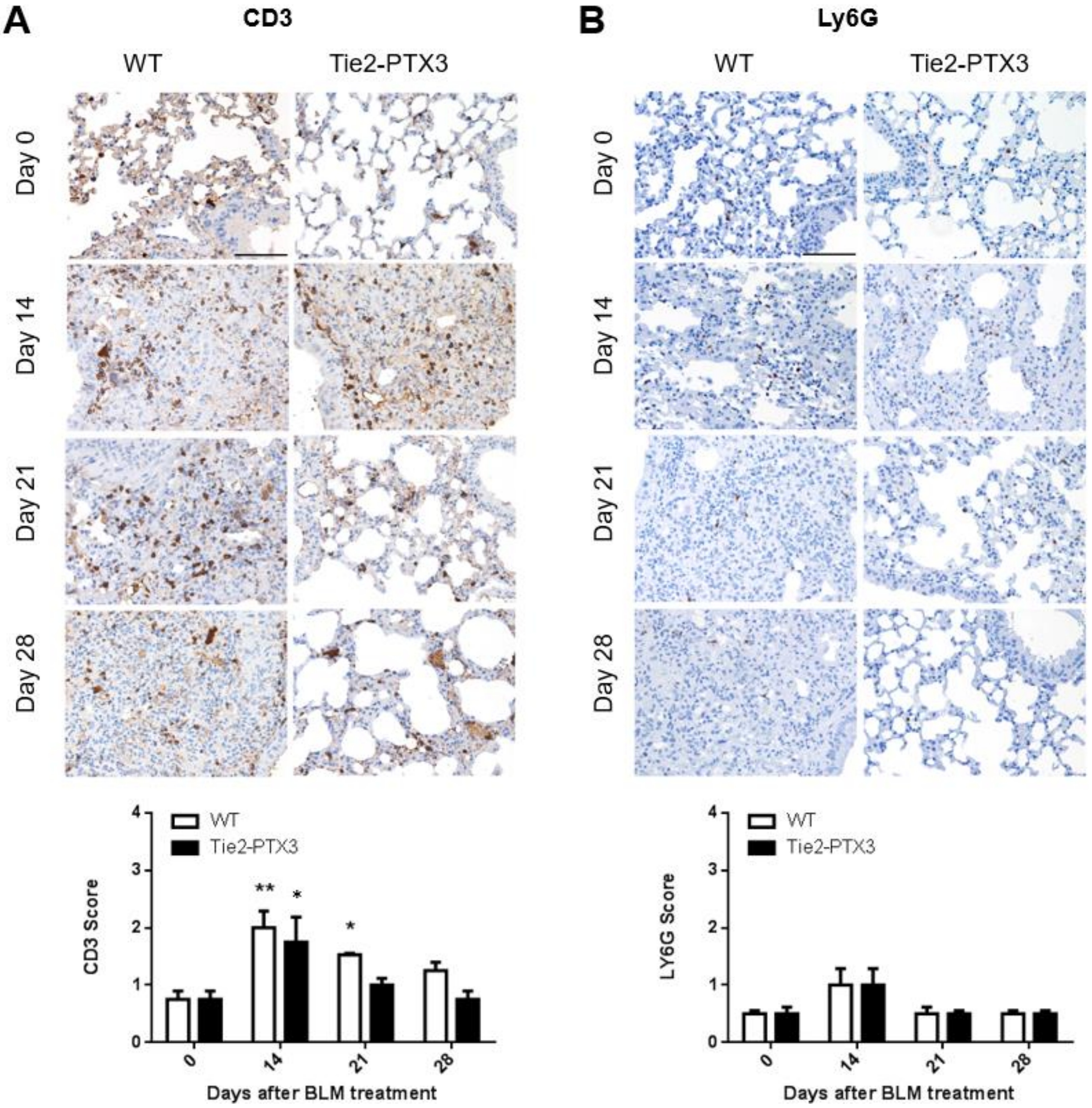

Supplementary Figure S4. Representative images and quantitative scoring for lymphocytes (**A**; CD3 staining) and neutrophils (**B**; Ly6G staining) performed on the lungs of BLM-treated WT and Tie2-PTX3 animals. *N* = 5-8 mice/group; scale bar = 100 µm; * P<0.05; ** p<0.01.
